# The Value of Automated Follicle Volume Measurements in IVF/ICSI

**DOI:** 10.3389/fsurg.2014.00018

**Published:** 2014-05-28

**Authors:** Frank Vandekerckhove, Victoria Bracke, Petra De Sutter

**Affiliations:** ^1^Centre for Reproductive Medicine, University Hospital Ghent, Ghent, Belgium

**Keywords:** automated follicle measurement, ovarian stimulation, three-dimensional ultrasound, *in vitro* fertilization, SonoAVC

## Abstract

**Background/Aims:** The objective of this literature study is to investigate the place of recent software technology sonography-based automated volume count (SonoAVC) for the automatic measurement of follicular volumes in IVF/ICSI. Its advantages and disadvantages and potential future developments are evaluated.

**Methods:** A total of 74 articles were read via a PubMed literature study. The literature study included 53 articles, 32 of which for the systematic review.

**Results:** The SonoAVC software shows excellent accuracy. Comparing the technology with the “golden standard” two-dimensional (2D) manual follicle measurements, SonoAVC leads to a significantly lower intra- and inter-observer variability. However, there is no significant difference in clinical outcome (pregnancy rate). We noted a significant advantage in the time gained, both for doctor and patient. By storing the images, the technology offers the possibility of including a quality control and continuous training and further standardization of follicular monitoring can be expected. Ovarian reserve testing by measuring the antral follicle count with SonoAVC is highly reliable.

**Conclusion:** This overview of previously published literature shows how SonoAVC offers advantages for clinical practice, without losing any accuracy or reliability. Doctors should be motivated to the general use of follicular volumes instead of follicular diameters.

## Introduction

The technology of ultrasound scans has undergone an enormous evolution since the early years of assisted reproductive technology (ART). Endovaginal ultrasound probes were developed during the 1990s and have replaced transabdominal ultrasound scans in ART (1). In addition to the improved resolution and image quality resulting from the proximity of the ovaries (2, 3), endovaginal ultrasound avoids the interruptive influence of abdominal fat, and it reduces the patient’s discomfort because, unlike transabdominal ultrasound, it does require a full bladder. The procedure is safe, and the examination time usually does not exceed 10–15 min. In practice, most doctors will estimate the follicular diameter based on the average of two or more individual readings. However, the measurements are highly subject to variation. Forman et al. (4) mention a trend of greater measurement errors in larger follicles. In addition, the reliability of follicular measurements decreases when the number of follicles increases (5). There is no consensus on how measurements should be performed, but it is plausible that with two-dimensional (2D) technology, a single measurement is less reliable than two or three measurements (6).

The development of three-dimensional (3D) ultrasound imaging in the late 1980s enabled the use and analysis of volume data (7). The unit constructing the image is now the voxel (defined by axes *x, y*, and *z*) rather than the pixel (defined by axes *x* and *y*) (1). 3D ultrasound imaging provides more accurate and reproducible volume measurements because the different image reproductions enable the investigator to detect surface irregularities and take corrective measures in the calculations. Because the difference between manual measurements in 2D and 3D proved to be modest and the first technologies for measuring 3D volumes were time-consuming, which limited their clinical applicability, manual 3D measurement techniques did not break through into clinical practice (8, 9).

**Figure 1:**
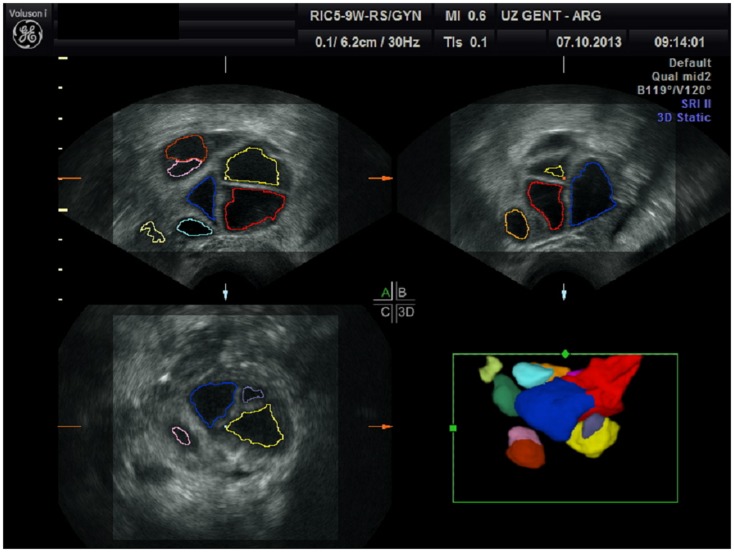
**Follicular measurement using sonography-based automated volume count (SonoAVC)**.

**Figure 2:**
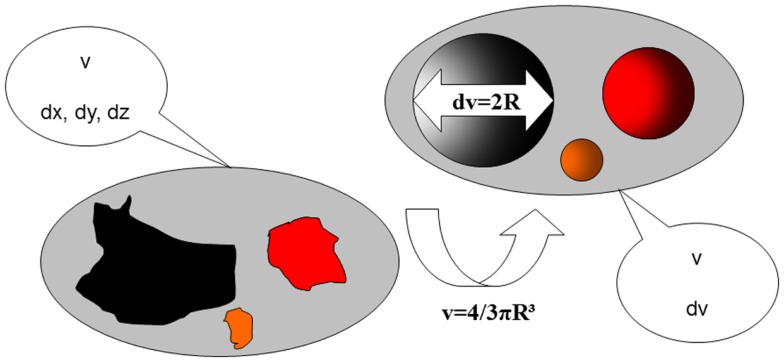
**The use of volume measurements compared to classical two-dimensional diameters of follicles**. *v*, follicular volume; d*x*, d*y*, d*z*, follicular diameters in three planes; *R*, follicular radius (diameter/2); d*v*, reconstructed follicular diameter.

Sonography-based automated volume count (SonoAVC; GE Medical Systems, Kretz, Austria) is a recently developed software program that automatically identifies the follicles in a specific ovarian volume and assesses their measurements (5). Because the follicles in the ovaries are filled with fluid, they are hypoechogenic structures within the relatively hyperechogenic ovarian tissue (7). SonoAVC identifies the hypoechogenic follicles within the selected ovarian volume, automatically analyses the volume data, identifies the boundaries of the follicles, and provides an estimate of their absolute measurements and volume (7, 10). The volume measurement is based on the amount of voxels within the identified follicle. Therefore, the measurement represents the actual volume of the follicle, regardless of its shape (7). In addition to a fully automatic analysis, SonoAVC provides post-processing options. For example, missed follicles can be added, and hypoechogenic structures wrongly identified as follicles can be removed (5). Automatic volume measurements require a 3D data set. This data set is represented via the multiplanar view, and a region of interest (ROI) is selected by manually moving a box in which the maximum proportions of the ovary fit (11). The frame selected by the ROI must be large enough to allow the entire ovary to be analyzed. Once this has occurred, SonoAVC software is applied to the data set. The automatic analysis lasts approximately 6 s; afterward, the individual follicles are represented, including their absolute and relative measurements (11). After the software has made automatic calculations of the entire ovary, the user can remove all extraovarian artifacts from the data set. SonoAVC software is either integrated into the ultrasound device or installed on a PC for the offline analysis of data sets obtained using an ultrasound device from the same manufacturer (12). Each volume has its own color (13), making SonoAVC an ideal tool for studying follicular development within the ovary (14) (Figure 1). In addition, the automatic measurements of the average diameter [mean follicular diameter (MFD)] of the follicle, its maximum measurements (*x, y, z* diameters), and its volume are represented from the highest to the lowest value (5, 13). The MFD is the arithmetic average of the longest three diameters in *x, y*, and *z* (7). Because SonoAVC is more accurate than a human investigator for determining the longest diameter in three orthogonal planes, this usually leads to higher MFD values (5). The *x, y, z* average diameter is calculated by taking the average of three perpendicular diameters. The software is developed to measure the maximum follicular diameter in the longitudinal or transverse plane first, followed by the two diameters perpendicular to it (11). The algorithm used to calculate the follicular volume (*V*) is based on the addition of all volume elements (voxels) within every hypoechogenic region (14). After recognizing the center of each structure, SonoAVC can calculate the exact number of surrounding voxels up to the edges of the structure and then extrapolate the average diameter and volume of the follicle (15). Theoretically, an unlimited number of volumes can be defined, making this technology ideal for follicle tracking (13, 16, 17). The volume-based diameter (*d*_v_) of the follicle is the diameter of a perfect sphere with the same volume as the follicle (7). The volume-based diameter *d*_v_, also referred to as the relaxed sphere diameter, is derived from the automatic volume measurement of the follicle. Using the relaxed sphere diameter formula (5), the diameter of the follicle is determined as if it were shaped like a perfect sphere (11) (Figure 2). It is normal for the values of the *d*_v_ measurements to be slightly lower than the manual measurements because the diameter of a perfect sphere will always be smaller than the average of three maximum diameters of an irregular follicle with the same follicular volume (MFD) (5). At present, ultrasound imaging is by definition a subjective interpretation of the image. Objective assessment of an ultrasound image requires a type of measuring system that must be reproducible (operator-independent) and must provide a valid result (11). Objective research assumes that assessments by different investigators (interobserver) and by the same investigator (intraobserver) will yield equivalent results; thus, objective research can be used to define standards and validate clinical practice (12).

This literature study investigates the role of automatic follicular measurements within the ART. This study mainly investigates the accuracy of automatic follicular measurements; thus, it must compare automatic follicular measurements with the gold standard, namely, manual follicular measurements with 2D ultrasound imaging. The intra- and inter-observer reliability of the new technology is evaluated in this study, as is the time gained and the clinical relevance of this method. In view of the high work pressure at fertility centers and the drive for an ever-higher treatment output, the application of quickly obtainable, reliable and reproducible data will gain importance in the future. Whether automatic follicular measurement technology contributes to this increased output remains to be seen. The value of the 3D technique for testing ovarian reserve by means of measuring ovarian volume (18) and antral follicle count (AFC) (19) is also investigated.

## Methodology

**Figure 3:**
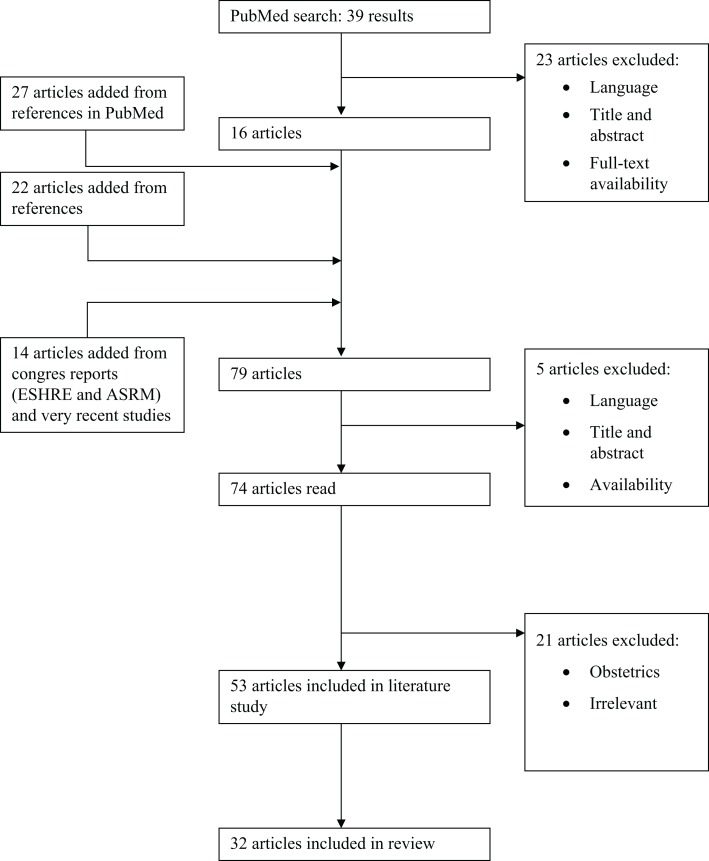
**Flow chart of the review process**. ESHRE, European Society of Human Reproduction and Embryology; ASRM, American Society for Reproductive Medicine.

The electronic database PubMed (1966–2014) was investigated (last updated April 19th 2014) using the following search terms: “automatic follicular measurement,” with 10 articles found; “SonoAVC,” with 35 results; “automatic volume measurement” and “follicle,” with 8 results. The search strategies supplied a total of 39 different articles. First, articles were excluded based on title and abstract, language (only English, French, and Dutch articles were included) and the availability of full-text articles. Sixteen articles remained. Next, the snowball method was applied to the reference lists of the included articles. Twenty-seven relevant studies were added to this literature overview. An additional 22 articles were added via the reference lists of these new articles. To enhance the effect of this literature study, the reports of the annual meetings from 2007 (the year SonoAVC was first applied) to 2013 of the European Society of Human Reproduction and Embryology (ESHRE) and of the American Society for Reproductive Medicine (ASRM were also checked for relevant articles. Fourteen abstracts were added in this way (Figure 3).

In total, 74 articles were fully studied. Nineteen articles were deemed unsuitable for this literature study because of their content and were excluded. Eventually, 53 articles were included in this literature overview. The articles were assessed according to their suitability for this literature study and their level of evidence. The studies comparing the new SonoAVC technology to previously existing 2D and 3D measurements (*n* = 32) were divided further into different categories of evidence: level A (randomized, double-blind, comparative clinical studies); level B (prospective studies without the characteristics of a level A or a retrospective cohort study); level C (non-comparative studies); and level D (experts’ opinion; Table 1). Because the new SonoAVC software for automatic volume measurements of an ultrasound data set was not developed until 2007, the articles describing and comparing this technology are very recent. A number of older articles were consulted for the history of ultrasound follicular measurements. Those articles do not counteract the topicality of this literature overview at all. The definitions used are from the glossary of the International Committee for Monitoring Assisted Reproductive Technology (ICMART) (20).

**Table 1:** **Systematic review of the literature**.

Study	Year	Type of study	Aims	Variables	Level of evidence
Ata and Tulandi (7)	2011	Review	Automated volume measurements in reproduction and pregnancy		B
Ata et al. (5)	2011	Prospective	Follicles were measured with 2D and SonoAVC in 100 patients	AFC	B
				Comparison of both techniques	
				Reproducibility	
Deb et al. (13)	2009	Prospective	AFC was measured with manual 2D, manual 3D, and SonoAVC in 55 patients	AFC	B
				Reproducibility	
				Intra- and interobserver reliability	
				Work flow	
Deb et al. (19)	2010	Prospective	AFC was measured with 2D and SonoAVC in 24 patients	AFC	B
				Work flow	
				Comparison of both techniques	
Deb et al. (24)	2013	Prospective	To evaluate intracycle variation in AFC	AFC of follicles ≤6 mm	B
Deutch et al. (31)	2009	Prospective	Evaluation of SonoAVC on phantom	Accuracy *in vitro*	B
			Offline analysis of 3D volumes with SonoAVC	Work flow	
Lamazou et al. (18)	2009	Review	Ultrasound in the evaluation of ovarian reserve		B
Lamazou et al. (15)	2010	Prospective	Aspirated follicle volumes of 72 follicles in 27 patients were compared with 3D volume measurements (VOCAL and SonoAVC)	Accuracy *in vivo* Comparison of both techniques	B
Murtinger et al. (46)	2009	Prospective RCT	The use of SonoAVC in the timing of oocyte maturation (hCG)	Number of (mature) oocytes Number of fertilizations	A2
			Comparison of 2D and SonoAVC		
				Pregnancy rates	
Raine-Fenning et al. (11)	2007	Conclusion	Evaluation of early studies	Accuracy	D
				Work flow	
				The importance of image quality	
Raine-Fenning et al. (12)	2008	Prospective	The aspirated volume of 224 follicles from 51 patients is compared with ultrasound measurements (manual 2D, VOCAL, SonoAVC)	Accuracy *in vivo* Comparison of techniques	B
Raine-Fenning et al. (6)	2009	Prospective	Follicle measurements with 2D and SonoAVC on the day of hCG triggering in 89 patients are compared	Comparison of techniques	B
				Work flow	
Raine-Fenning et al. (14)	2009	Prospective	The aspirated volume of 200 follicles is compared with ultrasound measurements (manual 2D, VOCAL, SonoAVC)	Accuracy *in vivo*	B
				Comparison of techniques	
				Work flow	
Raine-Fenning et al. (10)	2010	Prospective RCT	Comparing 2D and SonoAVC to define the moment of hCG triggering	Comparison of techniques	A2
				Number of (mature) oocytes	
				Number of fertilizations	
				Number of embryos	
Rodriguez-Fuentes et al. (17)	2010	Prospective	Follicle measurements with 2D and SonoAVC in 58 patients on the day of hCG triggering	The importance of image quality	B
				Work flow	
				The number of mature oocytes	
				Comparison of both techniques	
Rodriguez et al. (45)	2014	Prospective	To evaluate the number of procedures to reach proficiency in SonoAVC folliculometry	Learning curve–cumulative summation (LC–CUSUM)	B
Rousian et al. (32)	2009	Prospective	10 Phantoms studies with VOCAL, inversion mode, SonoAVC, and V-scope	Accuracy *in vitro* Intra- and interobserver reliability	B
			24 Gestational sacs in early pregnancy		
				Reproducibility	
Salama et al. (29)	2010	Prospective	Comparison of the aspirated volume with VOCAL and SonoAVC measurements in monofollicular cycles in 15 patients	Accuracy *in vivo*	B
				Comparison of techniques	
				Reproducibility	
Ata et al. (37)	2010	Prospective (OCA)	Comparing monitoring of COH with 2D and SonoAVC in 100 patients	Comparison of techniques	–
Bouhanna et al. (43)	2012	Prospective (abstract from ESHRE)	Comparing monitoring of monofollicular cycles with 2D, VOCAL, and SonoAVC in 22 patients	Comparison of techniques Reproducibility	–
Deutch et al. (38)	2007	Prospective (OCA)	Follicle measurements with 2D and SonoAVC of 10 follicles in three saved volume files by two observers	Comparison of techniques	–
				Intra- and interobserver reliability	
				Work flow	
Deutch et al. (42)	2007	Prospective (OCA)	Analysis of 347 follicles from 31 saved volume files in 14 patients	Work flow	–
				Reproducibility	
Hernandez et al. (13)	2009	Prospective (abstract from ESHRE)	Comparing monitoring with 2D, VOCAL, and SonoAVC on the day of hCG triggering in 27 patients	Association between follicle volume and number of mature oocytes	–
				Comparison of techniques	
Hernandez et al. (50)	2009	Prospective (OCA)	Comparing 2D and SonoAVC for monitoring oocyte donors	Comparison of techniques	–
				Number of (mature) oocytes	
				Number of fertilizations	
				Number of embryos	
				Implantation ratio	
				Pregnancy rate	
Hernandez et al. (40)	2011	Prospective (abstract from ESHRE)	Follicle measurements with 2D and SonoAVC in 100 patients	Work flow The importance of image quality	–
Raine-Fenning et al. (34)	2007	OCA	Follicles ranging 11 to 22 mm diameter were measured with 2D, manual 3D, and SonoAVC	Comparison of techniques	–
Raine-Fenning et al. (33)	2007	OCA	The aspirated volume of 100 follicles was compared with 2D, manual 3D, and SonoAVC measurements	Accuracy *in vivo*	–
				Comparison of techniques	
Rodriguez-Fuentes et al. (51)	2008	Prospective (OCA)	Monitoring with 2D, VOCAL, and SonoAVC in 46 patients	Comparison of techniques	–
				Association between follicle volume and number of mature oocytes	
Salama et al. (52)	2008	Prospective (OCA)	Comparing the aspirated volume with VOCAL and SonoAVC measurements in 15 patients with a monodominant follicle	Accuracy *in vivo*	–
				Comparison of techniques	
				Reproducibility	
Sanabria et al. (49)	2009	Prospective (abstract from ESHRE)	Monitoring with 2D or SonoAVC in 42 oocyte donor cycles	Comparison of techniques	–
				Number of (mature) oocytes	
				Number of fertilizations	
				Number of embryos	
				Implantation ratio	
				Pregnancy rate	
Saumet et al. (25)	2012	Prospective (OCA)	Comparison of different ultrasound techniques with AMH to measure ovarian reserve in 174 patients	Comparison of techniques	–
Sherbahn et al. (41)	2009	Retrospective (OCA)	Retrospective study comparing monitoring with 2D and SonoAVC	Comparison of techniques	–
				Work flow	

*OCA, oral communication abstract; ESHRE, European Society of Human Reproduction and Embryology*.

## Results

### Ovarian reserve testing

Antral follicle count determinations and measurements of the ovarian volume are easy to perform and inexpensive because ultrasound imaging facilities are readily available (13). In addition, ultrasound imaging is currently the only method that allows a direct assessment of each ovary as a separate entity (21). Ultrasound measurements of follicular numbers were developed as a “test” of reproductive age (22).

With total AFC determination (tAFC), the number of follicles between 2 and 10 mm is counted (21), usually on the third day of the cycle during the early follicular phase (23). The tAFC can be estimated using 2D or 3D ultrasound imaging (1, 16, 23). Recent reports have shown that more information can be obtained from observing the antral follicles than from quantification only (16, 24). The size of the antral follicles can actually be more significant than the total number of follicles and is therefore more predictive of the “functional ovarian reserve” (19). The success of ART treatment strongly relates to the number of antral follicles with a diameter between 2 and 6 mm, in terms of the relation of these follicles to the number of mature oocytes that can be collected. Deb et al. (24) were among the first to determine the AFC using SonoAVC software. With that software, they were able to identify and measure each antral follicle separately, an undertaking that would be very time-consuming and highly operator-dependent using 2D ultrasound imaging. Deb et al.’s studies (16, 24) support the theory that the smaller antral follicles actually reflect the ovarian potential and are most predictive of a response to controlled ovarian stimulation (COS). Following from that, a study by Saumet et al. (25) shows that 3D AFC determination and anti-Müllerian hormone (AMH) determination are equally capable of predicting the ovarian response, whereas 2D AFC determination is inadequate. This result can be explained by the fact that the smaller antral follicles (AFC if ≤6.00 mm) are mostly responsible for AMH secretion.

The ovarian volume can also be measured via transvaginal ultrasound (26) in the early follicular phase before any significant follicular dominance occurs (1, 23, 24). Studies by Lass et al. (3, 26) indicate that patients with very small ovaries (<3.0 cm) have to cancel the treatment cycle more often and have a significantly lower yield of oocytes; therefore, those patients have a lower chance of pregnancy. Ovarian volume measurement is fast and cost-effective, but its predictive value does not exceed that of tAFC (3, 26). In the study by Kupesic and Kurjak (27), total ovarian volume measured with 3D ultrasound imaging also showed a lower predictive value compared with tAFC. This difference can be explained by the fact that the number of smaller follicles (2–6 mm) decreases as a woman’s age increases, and the size of the follicles will slightly compensate for the decrease in tAFC as the age increases (28). Ovarian volume predicts the success of IVF to a lesser extent than tAFC does, but is useful in making a distinction between multifollicular and polycystic ovaries (27).

### Cycle monitoring and outcome of MAR

Assessing follicle size with 2D technology requires the measurement of every follicle in two dimensions and the calculation of the average diameter. This process is time-consuming, and the reliability of the measurements decreases when the number of follicles increases because it is difficult to check whether each follicle was measured once and only once (16). Because ovarian follicles often have complex shapes (29), particularly in stimulated ovaries with “follicular overpopulation” (1, 5, 12), manual determinations of the follicular diameter often offer a weak representation of the follicular volume and might have a negative effect on medical decision-making (17, 30). In these cases, volume measurement will provide more reliable information (1). These more precise results can lead to a higher follicle-to-oocyte ratio and a reduction in the number of immature oocytes (6). Therefore, it is assumed that 3D SonoAVC is closer to biological reality. Raine-Fenning et al. (14) studied 51 women undergoing COH for IVF therapy. The researchers measured the follicular volumes right before oocyte collection. The volumes obtained with SonoAVC were compared with the volume of the manually measured follicular aspirate. The actual follicular diameter was estimated by measuring the follicular volume using an aspirate and applying the formula 4/3πR^3^ to derive the diameter.

Raine-Fenning et al. (12) analyzed 224 follicles with an average follicular volume of 3.7 mL. SonoAVC measurements were very close to the actual follicular volumes, with an average difference of 0.04 mL (±0.25 mL). The study also showed that the 95% confidence interval is lower with SonoAVC compared with calculations based on one 2D-measured diameter (0.98 versus 5.88, respectively), which proves that SonoAVC is reliable. This study (12, 14) was the first to state that SonoAVC made the most accurate estimations of the follicular diameter compared with the actual follicular diameter. In this study, the *d*_v_ and MFD were of equal value. As in the *in vitro* studies (31, 32), these measurements seem to mildly underestimate the actual follicular diameter (maximum of 2.17 and 2.86 mm, respectively). Lamazou et al. (15) evaluated 27 women undergoing COS for IVF and compared the follicular volumes measured with SonoAVC to the actual volumes (based on the follicular fluid aspirate) of 72 follicles. The median actual volume was 3.20 mL (0.80–10.20 mL), compared with 3.25 mL (0.98–8.63 mL) for the volumes measured with SonoAVC. In another study, Raine-Fenning et al. (33) studied 100 follicles measured on the day of oocyte collection. Automatic volume measurements were compared with measurements of the follicular aspirate. Thus, the results showed that SonoAVC estimates the volume of a follicle most accurately using *d*_v_ results, with values that were almost identical to the estimated diameter based on calculations from the actual follicular volume (3.82 ± 3.03 versus 3.89 ± 3.07 mL, respectively). Automatic MFD calculations were less accurate (3.43 ± 2.75 mL) but still more precise than volume estimations of follicular diameters measured with manual 2D. Furthermore, the volume estimations made with manually measured diameters were more correct when 3D technology was used than when conventional 2D technology was used. In a similar study by Raine-Fenning et al. (34), five follicles of every diameter within a range from 11 to 22 mm were measured with SonoAVC using 2D manual measurements, 3D manual measurements and automatic volume measurements. In this case, the *d*_v_ values were also the most accurate ones.

Salama et al. (29, 35) studied 15 women undergoing IVF with embryo transfer in a cycle with a monodominant follicle. They measured the size of the dominant follicle before oocyte collection using SonoAVC software and performed the measurements three times. No significant difference was demonstrated between the three measurements using SonoAVC (3.57, 2.41–8.19 mL; 3.71, 2.49–8.90 mL; 4.07, 3.12–8.16 mL) and the actual follicular volume (3.60, 2.90–8.00 mL).

All of the studies published to date have independently shown that an obvious connection exists between follicular volumes calculated with SonoAVC and the actual follicular volumes obtained for follicular aspirate. Most of the studies showed that *d*_v_ values provided the most accurate result, but that MFD values provide more accurate results than manually measured 2D values do.

### Comparison of SonoAVC with manual 2D measurements

A significant question when comparing the two measurement techniques is how different the measurements are allowed to be without causing problems in clinical practice. Bland and Altman (36) investigated a way to measure the probability that a new technology differs from the old one. This method is the gold standard for comparing two measurement techniques.

Raine-Fenning et al. (6) studied 89 women undergoing IVF treatment and measured the number and average diameter of the follicles in both ovaries on day 10 using both 2D ultrasound imaging and SonoAVC with stored 3D volumes. The follicles were divided into different categories based on average diameter (≥10, ≥14, or ≥ 18 mm). A large degree of correlation existed between the two measurement methods (*r* = 0.84).

The average difference between both measurement techniques was less than 1 follicle in each category, demonstrating this technique provides very reliable results. Ata et al. (5, 37) studied 100 women undergoing COH after at least 5 days of stimulation. The average difference in the number of follicles measuring 14–17 and >18 mm between the 2D, SonoAVC-MFD, and SonoAVC-*d*_v_ measurements of the dominant follicle was less than 1 mm. In this study, SonoAVC-d_v_ resulted in slightly higher results than 2D manual measurements (on average, 1.3 additional follicles in the 10–13 mm category). On average, the difference in measurements decreased as the follicle size increased. The MFD measurements showed the same trend. On average, in follicles measuring 10–13 mm, more follicles (1.1) were detected with the SonoAVC technology. In this study, we also noted that the differences between both technologies increase when a woman has several follicles. Therefore, SonoAVC proves to create smaller *d*_v_ measurements because on average, the technology detects more follicles in the smaller categories. In comparison, on average, the technology detects fewer follicles in the largest category. Ata et al. (5), however, suggest that it is unlikely that the minor differences between the measurements would affect the clinical outcome. Deutch et al. (38) concluded after their study that no difference existed between the manually measured MFDs and the MFDs and *d*_v_s calculated using SonoAVC. In another study, Deutch et al. (31) studied 347 follicles in 14 women undergoing COH. They found the best correlation between the manually measured MFD and the *d*_v_ calculated with SonoAVC, with a correlation coefficient of 0.99. Rodriguez-Fuentes et al. (17) analyzed 92 ovaries in 58 women undergoing IVF treatment. They observed a significant difference in measurement results between conventional 2D technology and 3D technology with SonoAVC in 49% of ovaries (*n* = 45, *P* < 0.05). However, when they distinguished between good-quality and moderate- to poor-quality images, they observed proper correlation between 2D and SonoAVC in 62.3% of the good images (*n* = 33) versus 35.9% of the poor images (*n* = 14). In the study by Deb et al. (19), SonoAVC observed a significantly lower number of antral follicles than 2D ultrasound imaging did, both in tAFC and when the follicles were divided into five groups according to size. In the study, 24 women ≤40 years old who were undergoing ART were studied. After measurements of the AFC using the 2D mode, a 3D image was made of each ovary and SonoAVC was applied. The tAFC was significantly lower with SonoAVC (17.16 ± 9.71 versus 19.89 ± 10.33, respectively; *P* < 0.001). This result can demonstrate both an actual difference and a fundamental difference in the measuring techniques. Furthermore, the study showed that SonoAVC could demonstrate approximately half of the expected number of follicles with a diameter of 1–2 mm, while conventional 2D technologies were unsuccessful in registering one single follicle with these measurements. This difference most likely reflects the resolution limitations of the ultrasound imaging, but it can also be explained by the subjective nature of 2D ultrasound imaging. We noted the least similarity between the two measurement techniques for the antral follicles with a diameter of 3.0–4.99 mm. If the size of the follicles that contribute the tAFC is more important than the absolute size of the complete population – as suggested earlier – these findings have significant implications for clinical practice.

### Potential time gain with SonoAVC

With 2D ultrasound imaging, the investigator gradually rotates the transvaginal probe to scan each ovary while he/she identifies each follicle first and then measures its dimensions (39). This method is both time-consuming and uncomfortable for the patient (Table 2). However, a 3D examination only requires a quick view of each ovary while the probe registers the ultrasound data. The ovarian follicles can be counted and measured offline later. Various studies have evaluated the time required to apply SonoAVC.

**Table 2:** **Evaluation of work flow**.

	Manual 2D	SonoAVC	SonoAVC with post-processing (pp.)	2D versus SonoAVC with pp.
**IN COS CYCLES**				
Raine-Fenning et al. (6)	236.10 ± 57.07 s	39.00 ± 6.00 s	180.50 ± 63.55 s	*P* < 0.01
Rodriguez-Fuentes et al. (17, 40)	9.6 min	–	5.6 min	4 min time gain per patient
Deb et al. (19)	324.47 ± 162.22 s	41.06 ± 11.12 s	132.05 ± 56.23 s	*P* < 0.001
Deutch et al. (31)	361 s (129–878)	–	133 s (73–271)	*P* < 0.001, 7.6 min time gain per patient
Deutch et al. (38)	56.8 s	5.8 s		*P* < 0.001 without pp.
Sherbahn et al. (41)	6.2 min	–	5.6 min	
**IN SPONTANEOUS CYCLES**				
Raine-Fenning et al. (14)	26.36 ± 4.35 s	9.34 ± 0.67 s	–	*P* < 0.01 without pp.
**MEASURING tAFC**				
Deb et al. (13)	71 ± 8.48 s	26 ± 2.67 s	112 ± 29.86 s	*P* < 0.001

Raine-Fenning et al. (6) were the first to investigate time gained in stimulated cycles. In their study, 89 patients underwent an ultrasound scan on the 10th day of stimulation. The authors established that the ultrasound examination time required using the automatic 3D method was significantly shorter (*P* < 0.001) than examinations using conventional 2D technology (39.00 ± 6.00 versus 236.10 ± 57.07 s, respectively). The 3D technology required additional time for data analysis (141.50 ± 64.13 s), but the total time of 180.50 s (±63.55 s) was still significantly shorter than that associated with 2D technology use (*P* < 0.01). In all cases in the study, the ultrasound examination using 3D technology could be performed in <1 min, while the 2D manual measurement required approximately one additional minute.

Rodriguez-Fuentes et al. (17, 40) studied 58 women undergoing IVF and performed the ultrasound scan on the day of human chorionic gonadotropin (hCG) injection. They established that examinations of patients with >10 follicles lasted 9.6 min on average using conventional 2D technology, compared with 5.6 min using automatic monitoring. The examination was 7.6 min shorter on average for the patient with SonoAVC software. For the investigator, SonoAVC led to 4 min of time gained per patient. The study by Deb et al. (19) also showed that the average time of the automatic analysis using SonoAVC was significantly shorter than when 2D ultrasound monitoring was used (132.05 ± 56.23 and 324.47 ± 162.22 s, respectively; *P* < 0.001). The automatic analysis always required post-processing. The average time was 41.06 ± 11.12 s before the measurement and 90.99 ± 45.11 s before post-processing. In the study by Sherbahn et al. (41), a difference of 0.6 min on average was recorded (5.6 min for SonoAVC measurements and 6.2 min for 2D manual measurements). Deutch et al. (31, 42) also registered the time required to measure all follicles in an ovarian volume using both manual 2D measurements and SonoAVC. The differences in the number of follicles per ovary led to a large range of results. The time required for SonoAVC was significantly shorter than that required for manual measurements [133 s (range 73–271 s) versus 361 s (range 129–878 s), respectively; *P* < 0.001]. This led to savings of 3.8 min per ovary or 7.6 min per patient. With SonoAVC measurements, the post-processing took up the largest portion of time. Another study by Deutch et al. (38) only examined the measurement time (without analysis and post-processing). That study noted that the automatic software required 5.8 s on average to determine the diameter of all follicles, compared with 56.8 s for the manual method (*P* < 0.001).

### Investigation of operator dependence

Decreased operator dependence results in more reproducible folliculometry, as shown by low intraobserver and interobserver variability (Table 3).

**Table 3:** **Investigation of operator independence**.

		Manual 2D	SonoAVC	SonoAVC with pp.	*P*
**MEASURING tAFC**					
Deb et al. (13)		19.26 ± 10.55*	6.51 ± 4.79*	18.42 ± 10.53*	0.006**
		ICC = 0.979		ICC = 0.997	
**FOLLICLE MEASUREMENTS**					
Salama et al. (29)		ICC < 0.08	ICC = 0.97		
Bouhanna et al. (43)	Intra observer variability	ICC = 0.62	ICC = 0.96		
	Inter observer variability	ICC = 0.83	ICC = 0.94		

ICC, intra class correlation coefficient; *Mean ± SD; **Comparison of manual 2D and SonoAVC with pp.

Deb et al. (13) investigated 55 women with subfertility based on FSH levels <15 IU/L. Ultrasound scans were performed between days 2 and 5 of the menstrual cycle. The number of follicles in each ovary with a diameter between 2 and 10 mm was counted to determine the tAFC in that ovary. Three different methods were used to measure the number of follicles: 2D images in real-time (2D-RTE), 3D images in multiplanar view (3D-MPV), and 3D images in combination with SonoAVC software, whereby a distinction was made between those without (sAVC-AA) or with post-processing (sAVC-PP). These three methods were performed independently by two investigators. The median tAFCs measured using 2D-RTE, 3D-MPV, sAVC-AA, and sAVC-PP were 18 (10.56–26.81), 16.5 (10.25–26.75), 5 (3.37–9.18), and 15.5 (10.25–24.75), respectively. The initial tAFC investigated using SonoAVC without post-processing was missing a number of follicles, which is noticeable in its significantly lower median tAFC compared with all other methods (*P* < 0.001). The median tAFC after post-processing increased to 15.5, but remained significantly lower than the amounts found using 2D-RTE (*P* = 0.006) and 3D-MPV (*P* = 0.028). Calculations using Bland–Altman plots demonstrate that measurements using sAVC-PP are more reliable than measurements using 3D-MPV and 2D-RTE technologies are. Furthermore, with SonoAVC software, every data set was examined twice by the same investigator to assess the intraobserver variability. SonoAVC also provided the most reliable results after post-processing. Salama et al. (29) obtained an intraclass correlation coefficient (ICC) of 0.97 for the SonoAVC calculations in their study, indicating proper reproducibility. However, the manual measurements led to a lower ICC (<0.80). A study by Deutch et al. (38) also demonstrated that the intraobserver variability is significantly higher (*P* < 0.05) with the manual measurement of follicular diameters than with the automatic measurement using SonoAVC. The study by Bouhanna et al. (43) of 22 women in a monofollicular cycle also demonstrates that both the intra- (ICC = 0.96; 0.94–0.98) and interobserver (ICC = 0.94; 0.90–0.99) reliability of SonoAVC is higher than that of 2D manual measurements (ICC = 0.62; 0.45–0.79 and ICC = 0.83; 0.73–0.94, respectively).

### Possibility of standardization and new volume-based criteria for hCG triggering

A major problem with ART is the differences in pregnancy rates not just between different patients, but also between different centers. We notice differences between hospitals that cannot be explained by patient characteristics or regulations alone (44). Consequently, it seems plausible that the standardization of procedures could result in improved ART results. SonoAVC offers the possibility of standardizing follicular measurements so that scans of the ovaries can be performed by different investigators (5, 29). In addition, standardization opens the channel to more multicentre research (14).

Another advantage is the possibility of implementing a quality control system for follicular measurements. In practice, with 2D measurements, no data about follicular measurements other than the results can be kept. However, with SonoAVC, it is possible to store ovarian volumes. Such storage allows random checks to be performed on stored volumes and makes a quality control system available (5). Training programs show that a learning curve of 19 to 38 procedures is necessary to obtain proficiency in 3D SonoAVC (45). In addition, with SonoAVC, the quality standards drawn up by the European Union can be complied with because of the shorter examination time and the objective follicular diameter measurement (46).

Meseguer et al. (44) are convinced that the introduction of robotics in the ART laboratory can offer an advantage. Robotics can standardize various procedures and prevent interindividual differences. The new SonoAVC software offers a step in that direction.

Another important development that can be introduced via follicular volume calculations is the new volume-based criteria for hCG administration (5). Follicular volume calculation can provide a better indicator of oocyte maturation and increase the production of mature oocytes (5). The study by Salha et al. (47) shows this association is not simple. The authors were unable to relate the follicular volume to follicular readiness for fertilization and the possibility that the embryo will implant, despite the fact that in their study, embryo implantation ratios appeared to have decreased with follicular volumes of ≤1.0 or >5 mL. Rodriguez-Fuentes et al. (17) evaluated the relationship between the follicular volumes on the day of hCG administration and the number of mature oocytes obtained. The researchers wanted to establish cut-off values for the more exact timing of the hCG administration. In the study, 98.6% of the follicles ≥0.6 mL led to mature oocytes, while all other studied volumes overestimated or underestimated the number of mature oocytes. Because 3D measurements provide a better representation of the actual follicular size, the criteria for hCG in ovulation induction cycles, currently based on 2D technology, must be redefined. Murtinger et al. (46) established that during the last ultrasound scan, the average follicular diameter was lower with 3D (13.1 follicles) than with 2D imaging (15.1 follicles). This difference can be explained by the fact that 3D SonoAVC measurements measure more small follicles. That characteristic obviously raises the question of where the limit should be set to calculate the average. Because the values obtained with 2D and 3D are not sufficiently comparable in this regard, new criteria should be implemented that specifically match the more accurate measurements of SonoAVC. It is obvious that this new technology requires a complete revision of the criteria for hCG administration (30). Notwithstanding the fact that different studies dispute the improvement of the clinical result, the renewal of the criteria seems of interest. It was also very recently shown that the number of mature oocytes can be reliably predicted using volume measurements and that a distinction can even be made between different stimulation protocols (48).

## Discussion

What, then, is the actual value of automatic follicular measurements?

The discussed studies show that automatic measurements with SonoAVC estimate actual volumes with high accuracy. However, it offers no significant difference from the traditional 2D manual measurements, although an important annotation must be made here. In most of the abovementioned studies, the 2D measurements were performed by Clewes, an investigator with over 30 years experience in the manual measurement of follicular diameters (10). We can deduce from this that the automatic measurements provide results that are at least as accurate as those performed by an investigator with considerable expertise; furthermore, the automatic measurements are operator-dependent. Another annotation is the relatively high flexibility in the timing of the hCG administration and the oocyte collection, meaning that the (possibly) increased precision of the follicular measurements does not always result in an improved treatment result, such as an increased pregnancy ratio. These conclusions explain why to date, no improved clinical result can be demonstrated. Studies by Sanabria et al. (49) and Raine-Fenning et al. (10) demonstrate the lack of significant differences in clinical results between 2D manual measurements and SonoAVC (e.g., the duration of stimulation, number of oocytes, number of embryos, pregnancy ratio). The accuracy and clinical results therefore do not represent the actual value of the automatic measurements.

Sonography-based automated volume count software also has a few limitations, the most significant of which is the poor image quality obtained in some patients. According to the study by Rodriguez-Fuentes et al. (17), approximately 5% of patients cannot be monitored with the automatic technology, and another 15% require such intense post-processing that the automatic mode is not useful. Additional investigation into the influence of the patient’s BMI on the image quality seems of interest here, as we can assume that the amount of adipose tissue affects the image quality.

In addition, it is notable that according to a number of studies, SonoAVC provides underestimated measurements compared with manual 2D measurements. As demonstrated, these underestimations can be explained by the moderate to poor image quality of the ovary. After all, SonoAVC always looks for the inner definition of the follicle. Furthermore, possible “sound interference” in the follicle (as demonstrated in the *in vitro* studies) can lead to finding a lower number of voxels and therefore the calculation of smaller diameters. From this, we can conclude that SonoAVC is an automatic technology that still requires assessment by an investigator. This finding, combined with the required post-processing and the fact that 20% of the images must be analyzed manually, leads the classification of SonoAVC as a “semi-automatic” technology, as indicated by Ata and Tulandi (7) and Deb et al. (19).

The actual value of automatic follicular measurements, therefore, lies in the reliability of the measurements, on the one hand, and in the time gained with the technology, on the other. Both elements offer the clinician mostly logistic and educational advantages. Logistically speaking, the proven increase in intra- and interobserver reliability provides doctors the opportunity to have colleagues perform the measurements. In view of the low operator dependence, the various follicular measurements within a treatment cycle no longer have to be performed by the same doctor. Consequently, the IVF center can better distribute the tasks, and also the work pressure, among the various doctors. Furthermore, we noted that particularly in the stimulated cycles, both doctors and patients gain time. Doctors can see more patients within the same time span, which decreases the workflow at the IVF center. The patients also experience advantages: less-occupied waiting rooms, more time for communicating with the doctor, and less exposure to radio waves.

Regarding education, the use of 3D data and SonoAVC software can simplify the training of young doctors. On the one hand, a decreased learning curve for performing measurements is present (although it has not been investigated much in literature); on the other hand, we see how young doctors, investigators, and paramedics can be introduced to the (automatic) follicular measurements more easily via data storage capabilities. Furthermore this storage of images can lead to a quality control system via random checks to meet the ever-increasing (quality) requirements of patients.

Is it easy to implement automatic follicular measurements in clinical practice? SonoAVC is software installed on the ultrasound device or the analyzing computer and therefore requires minimal adjustment for the attending doctor. In addition, the doctor has the option of alternating the use of both 3D and 2D technologies. For example, clinicians will often use the manual 2D technology with monofollicular cycles and apply SonoAVC software with multifollicular cycles, when greater time-saving benefits are apparent. Furthermore, the transition from 2D manual measurements to automatic measurements is very user-friendly because clinicians can compare both technologies and thus learn to use the SonoAVC in folliculometry (31). However, upgrading the ultrasound devices with this new software involves additional costs. Furthermore, not all companies offer this option yet, which could restrict the possible use of SonoAVC (39). Therefore, further studies into cost-effectiveness seem appropriate. After all, it is difficult to measure the exact saving in terms of the decreased workflow at a fertility center.

When SonoAVC is used more often in clinical practice, new volume parameters are essential. Because not a single non-uniform 3D object really has an actual diameter, measurements of the follicular diameter remain arbitrary (11). As a consequence, volume parameters more accurately reflect reality. The development of new volume parameters can lead to the standardization of follicular measurements, possibly reducing the current differences among fertility centers worldwide.

Sonography-based automated volume count software does not offer any advantage in the clinical results of IVF treatment and has limitations in terms of poor image quality at times and the required post-processing after the automatic measurements. However, from this literature study, we can conclude that in approximately 80% of patients, the advantages of SonoAVC are very clear: the results are more reliable and therefore allow assessment by different (and relatively inexperienced) doctors; efficiency is increased because of saved time; and, in the future, SonoAVC will enable decisions based on follicular volume. It is hoped that the simplicity and accuracy of SonoAVC will help doctors transition from 2D to 3D measurements in the daily assessment of irregular structures, such as ovarian follicles (15, 29).

## Author Contributions

Frank Vandekerckhove and Victoria Bracke performed the literature search, analyzed the data, and wrote the article; they equally contributed to this work; Petra De Sutter critically revised the manuscript and approved it to be published.

## Conflict of Interest Statement

The authors declare that the research was conducted in the absence of any commercial or financial relationships that could be construed as a potential conflict of interest.
